# Comparison of traditional face-to-face teaching with synchronous distance education in medical theory courses teaching to medical undergraduates: A case-controlled study in China

**DOI:** 10.1097/MD.0000000000040714

**Published:** 2024-12-06

**Authors:** Xiaohong Lyu, Jun Zhao, Rui Tang, Hui Pan, Shi Chen

**Affiliations:** a Department of Endocrinology, Key Laboratory of Endocrinology of National Health Commission, Peking Union Medical College Hospital, Chinese Academy of Medical Sciences, and Peking Union Medical College, Beijing, China; b Department of Breast Surgery, Peking Union Medical College Hospital, Chinese Academy of Medical Sciences, and Peking Union Medical College, Beijing, China; c Education Division, Peking Union Medical College Hospital, Chinese Academy of Medical Sciences, and Peking Union Medical College, Beijing, China; d Department of Allergy, Beijing Key Laboratory of Precision Medicine for Diagnosis and Treatment of Allergic Diseases, National Clinical Research Center for Dermatologic and Immunologic Diseases, Peking Union Medical College Hospital, Chinese Academy of Medical Sciences, Peking Union Medical College, Beijing, China.

**Keywords:** case-controlled study, education, medical education, synchronous distance education, traditional education

## Abstract

Synchronous distance education (SDE) has recently been widely used for medical students. The purpose of this study was to compare the effects of SDE with traditional face-to-face education (TE) on the learning outcomes of undergraduate medical students. Participants were class enrolled in 2015 and class enrolled in 2016. The entire 2 classes (n = 148), when they were a 6th-year grade at a medical college, were invited to participate in the case-controlled study. The 6th-grade curriculum includes 5 courses in ophthalmology, otorhinolaryngology, dentistry, traditional Chinese medicine, and dermatology. Outcomes included postintervention exams evaluating the knowledge gained from the 5 courses, a self-assessment of learning based on examinable learning objectives, student satisfaction, learning behavior, and teacher–student communication. Sixty-nine students (SDE n = 39, TE n = 30) participated in the outcome assessments. The mean score of the otolaryngology course and the dermatology course in the SDE group was higher than the TE group (*P* < .05). Other courses and combined grades for the 5 courses were without statistical differences between the 2 groups. Of the 12 self-assessment learning objectives, 10 were significantly more positive in the SDE group than in the TE group. There was no statistical difference between the 2 groups regarding student satisfaction. The SDE group was more engaged than the TE group in the corresponding department apprenticeship after the theory class. The attendance rate and concentration in the class of the SDE group were comparable to the TE group. Students in the SDE group spoke more often in class and had a higher level of student–teacher communication than the TE group. The SDE is a teaching tool that can replace TE in the theoretical teaching of medical courses.

## 1. Introduction

Recent advances in network technology have improved synchronous distance education (SDE) in the form of online electronic learning. SDE simulates the communication model of traditional education (TE) by synchronizing teaching and learning, such as webcast conference rooms and virtual classrooms. Students and instructors must be present simultaneously for teaching to occur. It eliminates travel costs and time away from clinical training sites while still providing real-time interaction between students and instructors.

The use of SDE has received increasing attention in recent years, particularly in medical education.^[1–3]^ Many schools have attempted to offer SDE to medical students who are satellite campuses, internship hospitals away from the main campus,^[4–6]^ or to international students in different countries. Although previous studies have compared SDE with TE at the higher education level in medical education, their results have been inconsistent.^[4,7–9]^ A 2021 meta-analysis pooled previous studies on SDE.^[10]^ It pointed out that SDE was not significantly different from TE in effectiveness and had higher satisfaction ratings. Their finding might indicate the adoption of remote online education in medical education centers.

This study aimed to compare the effect of SDE with TE on the learning outcomes of undergraduate medical students. The secondary aims were to explore student satisfaction and evaluation. The study is the first case-controlled study in China to compare the effectiveness of online and offline medical education. The study can provide guidance and help for subsequent medical education approaches.

## 2. Methodology

This study was a case-controlled study among students with comparable learning bases and abilities. The experimental group used SDE in a semester-long medical education, and the control group used TE. Ethics approval for the study was obtained through the Peking Union Medical College Hospital Ethics Committee (S-K1990).

This study occurred over 2 years at Peking Union Medical College in 2021 and 2022. Participants were 6th year of 8-year clinical medicine education program students. The 6th-grade curriculum includes ophthalmology, dentistry, otorhinolaryngology, dermatology, and traditional Chinese medicine (TCM). Because of the localized COVID-19 outbreak in 2022, the 6th graders in 2022 went home to finish their medical education by SDE. However, the 6th graders in 2021 finished their education at school by TE. The 2 years of the 6th year cohort (n = 148) were invited to participate. Only students who complete the posttest questionnaire research will be included in all final analyses. Before this study, all students had 3 years of face-to-face clinical medical education. An independent research assistant recruited participants through an online explanatory statement and consent form.

The SDE environment utilized in this study was a real-time, interactive platform where students and teachers were online simultaneously. The platform featured webcast conference rooms that allowed for live lectures, screen sharing, and instant messaging. Virtual classrooms were equipped with interactive whiteboards, breakout rooms for small group discussions, and polling tools for immediate feedback. Teachers could present slides, demonstrate procedures, and engage students through Q&A sessions. Students could raise virtual hands, participate in discussions, and collaborate on shared documents. This environment aimed to replicate the interactivity of traditional classrooms while leveraging the benefits of digital tools.

The preceding semester grades were compared across the SDE and TE groups to test for the statistical difference at baseline. Use the previous semester’s diagnostic course grade as the baseline grade. A postintervention examination was administered immediately after the 6th-grade semester. The exams measured the knowledge level of 5 courses through the formal test on computers. All students in both grades took the exam. A month-long clinical apprenticeship followed the exams in the 5 courses for both groups. A postintervention survey was also administered after the 6th-grade semester. The survey measured the self-evaluation of participant demographics, knowledge mastery, perception of learning, learning behavior, course satisfaction, and communication degree of teachers and students. Most questions were measured for each examinable learning objective on a 5-point scale of strongly disagree to agree strongly. Data were collected in an online questionnaire under the supervision of the facilitator. Students voluntarily completed the posttest questionnaire research. Only those students who participated in the questionnaire research were eventually selected for this study. Exam results were also analyzed only for students who participated in the questionnaire study. The questionnaire is available in Appendix 1, Supplemental Digital Content, http://links.lww.com/MD/O127. The student survey was developed based on existing literature on medical education evaluation and was reviewed by a panel of 3 experts in medical education for content validity. The survey’s internal consistency was assessed using Cronbach alpha, which showed good reliability (α = 0.85).

All quantitative statistical tests were performed using Statistical Package for Social Sciences version 26.0 (SPSS Inc., Chicago, IL). Parametric quantitative data were reported using means and standard deviations. The unpaired Student *t* test was used to compare scores between the SDE and TE groups because the 2 groups are independent and our outcome variable (test scores) is continuous. The data in each group were normally distributed and that the variance was equal between the 2 groups. For the comparison of gender balance, Pearson chi-square test was used because gender is a categorical variable. *P*-values <.05 were as statistically significant.

## 3. Results

### 3.1. Participants

**Figure 1.**
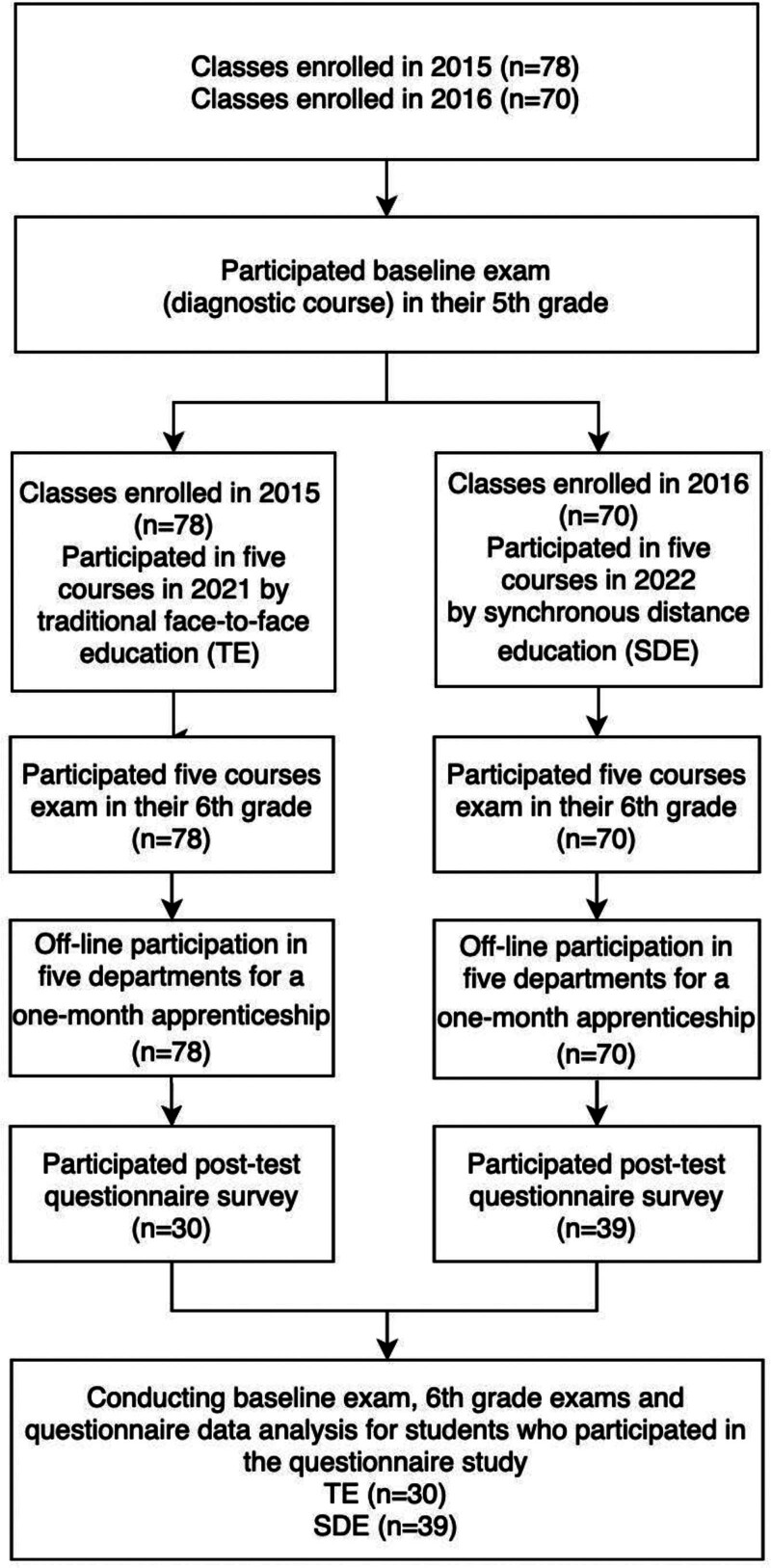
Flow-chart of student participation and data collection points.

Of 148 medical students, 69 (46.6%) completed the questionnaire. Sixty-nine students (TE n = 30 and SDE n = 39) participated in this study. Figure 1 is a summary of participation and data collection.

### 3.2. Baseline data

Means scores for the previous semester’s diagnostic course grade were comparable (SDE 84.62 ± 3.284, TE 83.80 ± 3.656; *P* = .327). The male-to-female ratio of the SDE and TE was 19:20 and 13:17, respectively, and were not significantly different (*P* = .657) (Table 1).

**Table 1:** Baseline data of 2 groups.

	SDE (n = 39)	TE (n = 30)	*P*-value
Previous semester’s diagnostic course grade (mean ± standard deviation)	84.62 ± 3.284	83.80 ± 3.656	.327
Sex (male)	19/39	13/30	.657

### 3.3. Postintervention knowledge exam for 5 courses

The 6th-grade curriculum includes 5 courses in ophthalmology, otorhinolaryngology, dentistry, TCM, and dermatology. All 5 courses have a perfect score of 100 points on the exam. The combined grade for the 5 courses is calculated based on the weighting of the 5 courses’ credit hours.

The mean score of the otolaryngology course in the SDE group was higher than the TE group, 88.14 ± 8.359 versus 77.50 ± 5.847, *P* < .001 (Table 2). The mean score of the dermatology course in the SDE group was higher than the TE group, 93.45 ± 2.947 versus 91.81 ± 2.310, *P* = .015. There was no statistical difference between the 2 groups’ scores in ophthalmology, dentistry, and TCM. The combined grade for the 5 courses (weighted by credit hours) was also without statistical difference between the 2 groups.

**Table 2:** Comparison of the subject scores of the 2 groups.

Courses (out of 100 points)	Mean ± standard deviation		*P*-value
	SDE (n = 39)	TE (n = 30)	
Ophthalmology	81.95 ± 4.482	82.04 ± 7.575	.952
Otolaryngology	88.14 ± 8.359	77.50 ± 5.847	<.001
Dentistry	85.15 ± 4.424	86.87 ± 5.191	.144
Traditional Chinese medicine	89.42 ± 6.490	91.57 ± 5.544	.151
Dermatology	93.45 ± 2.947	91.81 ± 2.310	.015
Combined grade for the 5 courses (weighted by credit hours)	87.70 ± 4.093	86.34 ± 3.963	.171

### 3.4. Self-assessment learning objectives

Of the 12 self-assessment learning objectives, 10 significantly differed between the SDE and TE groups (Table 3). Compared to TE classes, students in SDE classes have a higher self-assessment of their knowledge in all 5 courses.

**Table 3:** Comparison of the self-assessment of the 2 groups on the achievement of learning objectives.

Self-assessment of learning objectives achievement (1–5 points, 1 point for poor mastery, 5 points for good mastery)	Mean ± standard deviation		*P*-value
	SDE (n = 39)	TE (n = 30)	
The degree of knowledge of the clinical manifestations of glaucoma, cataract, and refractive diseases	4.38 ± 0.877	3.90 ± 1.125	.048
The degree of knowledge of the anatomy and physiology of the eye	4.41 ± 0.677	4.10 ± 1.029	.136
The degree of understanding of ophthalmic examination method	4.38 ± 0.782	3.97 ± 0.89	.042
Degree of knowledge of the anatomy of otology, rhinology, pharynx and laryngology	4.28 ± 0.826	3.10 ± 1.296	<.001
Degree of knowledge of clinical manifestations of common diseases such as otitis media, sinusitis and pharyngitis	4.21 ± 0.864	2.93 ± 1.23	<.001
Knowledge of laryngeal obstruction and tracheotomy	4.03 ± 1.063	3.13 ± 1.306	.003
The degree of understanding of the clinical manifestations of viral, bacterial and fungal skin diseases	4.51 ± 0.823	3.80 ± 1.126	.003
The degree of knowledge of the clinical manifestations and diagnostic points of major sexually transmitted diseases	4.46 ± 0.790	3.83 ± 0.913	.003
The degree of understanding of clinical manifestations of endodontic and periodontal diseases	4.31 ± 0.922	4.03 ± 1.159	.277
The degree of understanding of dental extractions and oral implantology related operations	4.13 ± 1.005	3.63 ± 1.033	.049
The degree of knowledge of traditional Chinese medicine (TCM) discriminative thinking, Chinese herbal medicine and prescription science	4.33 ± 0.898	3.70 ± 1.119	.011
The extent to your perception of TCM has changed	4.46 ± 0.969	3.80 ± 1.157	.012

### 3.5. Student satisfaction

The satisfaction of students in the SDE group and TE group are summarized in Table 4. Students were equally satisfied with the SDE and TE teaching. The SDE and TE teaching were equally helpful for exams and apprenticeships. Furthermore, the teaching approaches did not affect students’ career planning.

**Table 4:** Comparison of student satisfaction between the 2 groups.

	Mean ± standard deviation		*P*-value
	SDE (n = 39)	TE (n = 30)	
Are you satisfied with the teaching of these 5 courses (1 is the least satisfied, 5 is the most satisfied)	4.03 ± 0.986	3.77 ± 0.971	.280
Do classes help much in the exam (1 is the least helpful, 5 is the most helpful)	3.46 ± 1.315	3.33 ± 1.124	.671
How helpful are the classes for the apprenticeship? (1 is the least helpful, 5 is the most helpful)	3.67 ± 1.034	3.33 ± 1.093	.200
Do you have the desire to become a doctor in one of these specialties in the future (1 least wanted, 5 most wanted)	2.85 ± 1.479	2.47 ± 1.432	.288

### 3.6. Learning behavior

The SDE group was more engaged than the TE group in the corresponding department apprenticeship after the theory class (1–5 self-assessment points, SDE 4.26 ± 0.818 vs TE 3.43 ± 1.104, *P* = .001) (Table 5). The attendance rate and classroom concentration of the SDE group were comparable to the TE group.

**Table 5:** Comparison of the learning behaviors of the 2 groups of students in the course.

	Mean ± standard deviation		*P*-value
	SDE (n = 39)	TE (n = 30)	
Your weekly absence frequency for online or offline classes is (1 is full attendance, 5 is full absence)	1.54 ± 0.913	2.00 ± 1.145	.067
How often do you complete other tasks in class at the same time? (Other tasks include: study in other subjects, research tasks or mobile games, etc) (1 is focused, 5 is always doing tasks that are not related to the course)	2.26 ± 1.033	2.37 ± 1.033	.664
How often do you follow the instructor during class and listen to the whole course? (1 is to listen to the whole course, 5 is not to listen at all)	2.15 ± 1.182	2.50 ± 1.137	.225
Your participation in the apprenticeship in the corresponding department after taking the theory course (1 point participation is the lowest, 5 points participation is the highest)	4.26 ± 0.818	3.43 ± 1.104	.001

### 3.7. Communication degree of teachers and students

Students in the SDE group spoke more often in class (with more passive questions) and had a higher level of student–teacher interaction (Table 6) than the TE group. There was no difference between the 2 groups in the number of student-initiated questions.

**Table 6:** Comparison of the level of student–teacher communication between the 2 groups.

	Mean ± standard deviation		*P*-value
	SDE (n = 39)	TE (n = 30)	
What is the total number of times you speak in class each week? (1 being 0 times, 5 being more than 5 times)	1.74 ± 0.498	1.30 ± 0.535	.001
The total number of times you ask questions with your teacher each week is (1 is 0 times, 5 is more than 5 times)	1.41 ± 0.498	1.33 ± 0.547	.544
Do you think the teachers have a high level of interaction with the students in teaching the 5 senses? (A score of 1 is extremely high, 5 is no interaction)	2.23 ± 0.583	2.93 ± 0.868	<.001

## 4. Discussion

Our study showed no significant difference between the SDE and TE groups regarding their combined exam scores. The questionnaires showed that the SDE group was better than the TE group in learning goal attainment, learning behavior, and teacher–student communication. This result corresponds with findings from He et al meta-analysis.^[10]^ Their study showed no significant differences between SDE and TE in objective assessments, and SDE was more acceptable in subjective evaluations than in traditional education.

In our study, the overall subjective evaluation of questionnaire research was higher in the SDE group than in the TE group. There might be some possible reasons for the positive subjective evaluations of SDE. Firstly, SDE gives students more freedom to study. Students can complete their online studies from any location they choose. The saved commuting time is more effectively allocated to student research or recreational time. As a result, students do not spend time in class doing tasks other than studying, and course focus is increased (Table 5). Secondly, students can record their lessons and quickly review what they do not understand. This point may also improve students’ exam performance and learning objective achievement in some of the courses, for example, the otolaryngology course (Tables 2 and 3). Thirdly, students can review the online course content anytime they encounter a problem they do not understand during their apprenticeship. This may have increased students’ engagement in the SDE group during their departmental apprenticeship (Table 5). Finally, the higher frequency of student–teacher communication in the SDE group may be due to the teacher’s compensatory questioning. Since teachers and students cannot face each other, many teachers will worry that there is insufficient teacher–student communication. So instead, teachers will ask additional questions in the SDE group to facilitate communication. We can see in Table 6 that the mean number of student-initiated questions does not differ significantly between the 2 groups (1–5 points, SDE 1.41 ± 0.498 vs TE 1.33 ± 0.547, *P* = .544).

This study is the first case-controlled study that identified the effectiveness of SDE compared to TE for medical students in China. More importantly, this study meets a current critical demand for medical education due to the COVID-19 pandemic. Due to the unprecedented COVID-19 pandemic, many medical colleges have been forced to close classrooms and quickly convert to SDE. SDE is the most adopted option for health science students to maximize online communication and interaction with their instructors because it provides real-time interaction and synchronized communication between students and instructors. Concerns about the quality of education may be somewhat mitigated given that there is no significant difference in theory teaching effectiveness between SDE and TE.

Recent advancements in technology have also addressed concerns about student engagement in distance education. Ozdemir and Ugur (2021)^[11]^ proposed a model using face recognition technology to determine student attendance in distance education. Their study highlights the potential for technological solutions to monitor and potentially improve student participation, attention levels, and teacher–student interaction in online learning environments. This aligns with our findings on comparable attendance rates and classroom concentration between SDE and TE groups, and the higher level of student–teacher communication observed in the SDE group.

Despite the valuable results of this study, there are some limitations. First, conducting randomized controlled trials within an educational context is difficult. Students enrolled in 2016 had to be taught by SDE in 2022 because of the COVID-19 pandemic. As a result, we performed a case-controlled study. Future studies addressing newly developed SDE technologies and large-scale randomized controlled trials about SDE are required to verify this study’s results further. Second, since the questionnaire was voluntary, only students who participated in the questionnaire were included in the study. This may lead to selection bias among participants. Perhaps those students who felt better about themselves or had better grades participated in the questionnaire study. However, this voluntary participation in the study was implemented equally in the SDE and TE groups to minimize bias. Finally, the courses involved in this study were all theoretical teaching components of the respective sections. Therefore, all conclusions apply to theoretical teaching only. For the practical teaching section, all students were still taught by TE. While this study focused on theoretical courses, the potential application of SDE in practical medical education warrants further investigation. Future studies could explore the use of virtual simulations, augmented reality, and remote observation techniques in teaching practical skills. A hybrid model combining SDE for theoretical preparation with targeted in-person sessions for hands-on practice might optimize learning outcomes in practical courses. However, challenges such as ensuring proper skill acquisition, maintaining patient privacy, and adapting assessment methods need to be carefully addressed in such implementations.

Future studies could explore the integration of virtual reality (VR) technology in medical education, both in distance and traditional settings. As demonstrated by Ozdemir and Ozturk^[12]^ in geography education, VR applications can significantly impact academic achievement and student interaction. In the medical field, VR could potentially enhance practical skills training and anatomical understanding, offering new avenues for both SDE and TE.

## 5. Conclusion

Our study’s SDE group was no worse than the TE group in test scores. Moreover, the subjective evaluation of the questionnaire in the SDE group was more positive than the TE group in terms of teaching goal achievement, learning behavior, and teacher–student communication. Therefore, SDE teaching is a tool that can replace TE teaching in the theoretical teaching of medical courses.

## Acknowledgments

We would like to express our sincere gratitude to every student who participated in the survey.

## Author contributions

**Conceptualization:** Rui Tang, Shi Chen, Hui Pan.

**Data curation:** Jun Zhao.

**Funding acquisition:** Hui Pan.

**Writing – original draft:** Xiaohong Lyu.

**Writing – review & editing:** Hui Pan.

## Supplementary Material


