# Supplementary material for: Comparison of traditional face-to-face teaching with synchronous distance education in medical theory courses teaching to medical undergraduates: A case-controlled study in China
Source: Medicine (Baltimore). 2024 Dec 6;103(49):e40714. doi: 10.1097/MD.0000000000040714 (PMC11630938; doi:10.1097/MD.0000000000040714)
Supplement: Supplementary file 1 [file medi-103-e40714-s001.docx]

**Appendix 1：**

**Online/offline teaching effectiveness evaluation questionnaire for the second semester of the sixth grade**

Hello, dear students, the teachers of the Teaching Committee of the Peking Union Medical College would like to survey the evaluation of the effectiveness of the online/offline teaching of medical students in the second semester of the sixth grade, which involves the teaching of five courses: ophthalmology, otorhinolaryngology (ENT), dentistry, dermatology and traditional Chinese medicine (TCM). The teachers will adjust the future teaching mode according to the teaching evaluation to achieve a better teaching effect. Please fill out the questionnaire; it only takes about 3 minutes~

Please note that.

1. the questionnaire participation is anonymous.

2. The questionnaire is voluntary.

3. Any answers will not affect current or future assessments.

4. You can opt out of the survey at any time; we guarantee that doing so will not affect your results.

# Baseline information

1. The teaching method to complete ophthalmology, ENT, dermatology, dentistry, and TCM is?

A. Online (Tencent conference)

B. Offline (in the classroom)

2. Gender

A．Male

B．Female

3. Your major and grade

A．Eighth-year class of 2015

B．Eighth-year class of 2016

# Course Awareness

Please rate how well you have accomplished the specific teaching objectives for each of the five courses on a scale of 1-5, with 1 being the lowest and 5 being the highest.

**Section 1-** **Ophthalmology (1-5 points, 1 being the lowest and 5 being the highest)**

1. The degree of knowledge of the clinical manifestations of glaucoma, cataract, and refractive diseases

2. The degree of knowledge of the anatomy and physiology of the eye

3. The degree of understanding of the ophthalmic examination method

**Section 2 - Otolaryngology (1-5 points, 1 being the lowest, 5 being the highest)**

1. Degree of knowledge of the anatomy of otology, rhinology, pharynx and laryngology

2. Degree of knowledge of clinical manifestations of common diseases such as otitis media, sinusitis and pharyngitis

3. Knowledge of laryngeal obstruction and tracheotomy

**Section 3-** **Dermatology (1-5 points, with 1 being the lowest and 5 being the highest)**

1. The degree of understanding of the clinical manifestations of viral, bacterial and fungal skin diseases

2. The degree of knowledge of the clinical manifestations and diagnostic points of major sexually transmitted diseases

**Section 4-** **Dentistry (1-5 points, 1 being the lowest and 5 being the highest)**

1. The degree of understanding of clinical manifestations of endodontic and periodontal diseases

2. The degree of understanding of dental extractions and oral implantology-related operations

**Section 5-** **Traditional Chinese Medicine (1-5 points, 1 being the lowest and 5 being the highest)**

1. The degree of knowledge of TCM discriminative thinking, Chinese herbal medicine and prescription science

2. the extent to your perception of TCM has changed

# Learning behavior

1. How often do you miss classes online or offline each week?

A．Full attendance, never missed a class

B. Occasionally absent once

C. 2-3 times a week

D．Absence 4-5 times a week

E. Most of the time

2. How often do you complete other tasks while in class? Other tasks include a study of other subjects, research tasks or mobile games, etc.

A. Rarely (always paying attention to class)

B. Occasionally (less than 1/3 of the class is spent on other tasks)

C. Fairly (about 1/2 of the course is completing other tasks)

D. Frequent (more than 2/3 of the lessons are on other tasks)

E. Very frequently (almost every class is on another task)

3. How often do you follow the teacher in class?

A．Tried to listen to the whole course in every lesson

B．Listen to the whole course in more than 2/3 of the classes

C．Listen to the whole course in about 1/2 of the classes

D．Less than 1/3 of the lessons are listened to completely

E. Rarely listen to the whole course

# Degree of communication between teachers and students

1. The total number of times you speak in class each week?

A. 0 times B. 1-2 times C. 3-5 times D. more than 5 times

2. The total number of times you ask questions with your teacher each week?

A. 0 times B. 1-2 times C. 3-5 times D. 5 times or more

3. Do you think the teachers interacted well with the students in the second semester of 6th grade?

A. Very high, the teachers were active in asking questions and even gesturing and distributing teaching materials to demonstrate

B．High, the teacher actively asked questions

C. Fair, the teacher asked and answered questions by himself/herself

D．The teacher finished the lecture and left

E．The teacher only read the PPT

# Knowledge mastery

1. What primary materials are you referring to when preparing for the exam?

A. The notes were taken in class and the teacher's slides

B．Textbooks

C. Notes left behind by older students in previous years

D. Other teaching materials

E. Other materials

2. How helpful do you think the online/offline courses in the second semester of 6th grade were for the exam? (On a scale of 1-5, 1 being the lowest and 5 being the highest).

3. How helpful do you think the online/offline courses in the second semester of 6th grade were for apprenticeships in the respective department? (On a scale of 1-5, 1 being the lowest and 5 being the highest).

4. What was your level of participation during your apprenticeship in the corresponding department? (On a scale of 1-5, with 1 being the lowest and 5 being the highest)

# Course Satisfaction

1. Are you satisfied with the teaching of ophthalmology, otorhinolaryngology, dentistry, dermatology, and Chinese medicine in the second semester of the sixth grade? (On a scale of 1 to 5, with 1 being the lowest and 5 being the highest)

2. the extent to which the course will help in the future practice of medicine (1-5 points, with 1 being the lowest and 5 being the highest)

3. the desire to become an ophthalmologist/ ENT/ dermatologist (1-5 points, 1 being the lowest and 5 being the highest).

4. If you could choose, how would you like to complete the five courses?

A. Online

B．Offline
